# Diagnostic Dilemma of Disseminated Histoplasmosis Mimicking Hemophagocytosis Lymphohistiocytosis in Patient with Rheumatoid Arthritis on Anti-TNF Therapy: Case Report and Review of the Literature

**DOI:** 10.1155/2019/4169052

**Published:** 2019-02-12

**Authors:** Saurav Suman, Aleksander Lenert

**Affiliations:** ^1^Assistant Professor, Division of Hospital Medicine, University of Kentucky, Lexington, USA; ^2^Assistant Professor, Division of Rheumatology, University of Kentucky, Lexington, USA

## Abstract

Tumor necrosis factor inhibitors (TNFi) have become the cornerstone for the treatment of rheumatoid arthritis and other systemic autoimmune conditions. However, these biologic DMARDs can lead to various opportunistic infections such as viral infection, tuberculosis, and histoplasmosis. Furthermore, these biologics can also cause severe systemic inflammatory reactions known as hemophagocytosis lymphohistiocytosis (HLH) that can lead to multiorgan failure and high mortality. Due to overlapping clinical features and time-intensive microbiological culture methods, distinguishing between HLH and opportunistic infections can be challenging early in the disease course. We present a similar situation with our patient where the patient met the diagnostic criteria for HLH however was found to have disseminated histoplasmosis. This case uniquely evaluates the utility of the HLH diagnostic criteria and hemophagocytosis for accurate diagnosis of HLH.

## 1. Introduction

Patients with rheumatoid arthritis who are on immunosuppressive medications such as tumor necrosis factor inhibitors (TNFi) are at increased risk for developing infections. Fever and cytopenia should warrant a prompt investigation as the differential diagnosis ranges from self-limiting viral infections to disseminated invasive infections such as tuberculosis and histoplasmosis.

## 2. Case Presentation

A 63-year-old Caucasian female with established seropositive (+RF, +ACPA) rheumatoid arthritis for 11 years on treatment with infliximab and methotrexate (MTX; 20 mg/week) presented with a 1.5-month history of fever, cough, and dyspnea. She was initially suspected to have community-acquired pneumonia treated with antibiotics and systemic steroids with transient improvement; however, her symptoms recurred. Initial lab tests showed anemia (Hb: 8.8) and thrombocytopenia (platelet: 51) with an elevated ferritin of 28,000 ng/ml, low complements (C3: 21; C4: <2), low fibrinogen, elevated CRP, normal lipids, and mildly elevated liver enzymes. CT chest was remarkable for subpleural opacity without parenchymal infiltrates. Her physical examination demonstrated chronic limited range of motion of the left wrist and no active synovitis.

Due to significant elevation in ferritin, recurrent fevers, cytopenias, and failure to respond to antibiotics, secondary hemophagocytic lymphohistiocytosis (HLH) was suspected, with possible triggers being RA, immunosuppression, malignancy, and infection. Additionally, further testing demonstrated an elevated soluble IL-2 receptor (sIL-2) at 7970 U/ml (Ref: 45–1105 U/ml). Hence, the patient fulfilled 5 out of 8 HLH-2004 diagnostic criteria for HLH with elevated ferritin, low fibrinogen, fever, cytopenia (Hb < 9 and low platelets), and elevated sIL-2 receptor. She was empirically started on high-dose systemic glucocorticoids for management of HLH; however, she continued to experience recurrent high-grade fever. Due to suspicion for an underlying infectious etiology of HLH, bone marrow biopsy was performed which revealed hypercellular bone marrow with multiple fungal elements consistent with histoplasmosis without evidence of hemophagocytosis. Subsequent urine and blood cultures confirmed disseminated histoplasmosis.

Antifungal treatment with intravenous amphotericin was promptly initiated in the hospital; after adequate clinical response, the patient was transitioned to itraconazole. Infliximab and MTX were held during antifungal treatment, and the patient was switched to hydroxychloroquine. Her infection was under control during her 9 months of follow-up with infectious disease, and she remained on antifungal treatment indefinitely.

## 3. Discussion

Biologic disease-modifying antirheumatic drugs (DMARDs) have become the cornerstone for treatment of systemic autoimmune disorders. With the advancement in technology and research, new biologics are emerging that help improve the treatment and prognosis of autoimmune conditions. However, these great benefits are not without significant risks. One of the important side effects associated with the use of biologics is the risk for development of opportunistic infections (OIs). Different classes of biologics differ in their ability to cause OIs. Certain tumor necrosis factor inhibitors (TNFi) such as infliximab and adalimumab are associated with higher risk of OI compared to etanercept [1]. According to a recent meta-analysis of RA patients on biologics, for every 1000 patients treated with biologics, 1.7 patients developed an OI (0.0017%) [2].

TNFi can lead to OIs due to direct inhibition of tumor necrosis factor (TNF). TNF is essential for the formation of granuloma and prevention of granulomatous infections such as tuberculosis and histoplasmosis. Histoplasmosis is the most common fungal OI with an estimated incidence of 18.78 per 100,000 persons (0.00018%), second only to tuberculosis with an incidence of 53.81 per 100,000 (0.00053%) patients treated with infliximab [3].

Histoplasmosis is a fungal infection that primarily involves the lungs. Symptoms associated with acute pulmonary histoplasmosis include fever, chills, cough, and dyspnea that are similar to bacterial pneumonia. Mediastinal adenopathy, which is common in histoplasmosis, can lead to pleuritic chest pain. Once the infection spreads systemically, it targets several organs including the liver, spleen, gastrointestinal tract, and bone marrow. Immunocompromised state, due to HIV or anti-TNF therapy, increases the risk of systemic infection with histoplasmosis [4]. Approximately 75% of histoplasmosis cases associated with TNFi have disseminated disease with a mortality rate of 3.2% [5]. Additional symptoms noted during the disseminated phase include fever, chills, fatigue, weight loss, and anorexia. Physical examination typically reveals lymphadenopathy and hepatosplenomegaly. Laboratory investigations are significant for pancytopenia, transaminitis, hyperbilirubinemia, elevated lactate dehydrogenase, and hyperferritinemia [6]. The diagnosis is confirmed by urine and serum testing for histoplasma antigen, as well as cultures from bronchoalveolar lavage (BAL) for definitive microbial identification and cytopathology [4]. However, these batteries of diagnostic tests are time- and resource-consuming, and final confirmatory results are often delayed. Due to clinical symptoms overlap of disseminated histoplasmosis with hemophagocytic lymphohistiocytosis (HLH), timely additional workup is needed in suspected cases of HLH to distinguish between the two, as the mortality associated with untreated HLH can exceed 50% [7].

HLH should be considered in any patient with persistent fever, cytopenia, transaminitis, and hyperferritinemia [8]. These findings can overlap with those of invasive histoplasmosis. RA patients on TNFi are at risk for HLH [9]; however, there are no reported cases of HLH in RA patients not on biologic DMARD. In contrast, both pulmonary histoplasmosis and disseminated histoplasmosis have been reported in RA patients irrespective of TNFi use [10]. Interestingly, there have been several reports of histoplasmosis associated HLH infection dating back to 1993 [11].

HLH is characterized by widespread multiorgan inflammation that involves prolonged activation of antigen presenting cells (i.e., macrophages and histiocytes) and CD8+ T cells, in response to an antigen trigger. Natural killer (NK) cells, which function to attenuate the inflammatory signal, are found to be functionally deficient in HLH [12]. The prolonged activation leads to excessive formation of cytokines (“hypercytokinemia”) and multiorgan failure [12].

HLH is diagnosed using the HLH-2004 diagnostic criteria. Traditionally, HLH was classified into primary form (usually in kids; documented genetic changes and abnormal function of NK and cytotoxic T cells) and secondary form, HLH mostly seen in adults (HLH features in the presence of a trigger such as infection, without the genetic changes). However, the genetic changes can be seen in adults as well, and thus, primary and secondary forms of designations have now been corrected to “genetic” and “acquired” forms of HLH [13].

HLH can be diagnosed either with

1. genetic testing to check for molecular arrangement consistent with primary HLH or
2. 5 out of 8 of the following findings [14]:
  - Fever
  - Splenomegaly
  - Cytopenia: affecting 2 of 3 cell lines in the peripheral blood (hemoglobin in adults < 10 mg/dL, platelets <100 ∗ 10^9^/L, and neutrophils 1 ∗ 10^9^/L)
  - Hypertriglyceridemia (≥265 mg/dl) and hypofibrinogenemia (<1.5 g/L)
  - Hemophagocytosis in bone marrow, liver, or spleen with no evidence of malignancy
  - Absence of or low natural killer (NK) cell activity (local lab reference)
  - Ferritin > 500 microgram/L
  - Soluble CD 25 (i.e., soluble IL-2 receptor) ≥ 2400 U/ml

Hemophagocytosis on bone marrow biopsy is an important feature of HLH and considered the gold standard for diagnosis. In hemophagocytosis, erythrocytes, leukocytes, thrombocytes, and their precursors are engulfed by macrophages [15]. In addition to HLH, hemophagocytosis can be seen on tissue biopsy in the setting of infection, bone marrow hyperplasia, ineffective hematopoiesis, major surgery, blood transfusion, and chemotherapy. There is an ongoing debate regarding the role of hemophagocytosis in the diagnosis of HLH. A study evaluating 80 bone marrow biopsies with hemophagocytosis concluded that pancytopenia and high-grade hemophagocytosis (defined as severe >5 histiocytes with hemophagocytosis/slide) may help distinguish between HLH and non-HLH etiologies [16]. In contrast, another recent study analyzing 64 bone marrow biopsies from 58 patients concluded that hemophagocytosis does not reliably predict HLH [17].

## 4. Conclusion

This patient met the diagnostic criteria for HLH; however, the bone marrow biopsy did not show hemophagocytosis. Her treatment for HLH was initiated with immunosuppression without any response. Subsequently, she was switched to antifungal therapy for disseminated histoplasmosis and showed excellent response. In the current diagnostic criteria of HLH, presence or absence of hemophagocytosis does not mandate or refute the diagnosis nor does it have any prognostic significance. However, this case was successfully treated with antifungal alone without the need for additional immunosuppression. It is worth exploring whether the absence of hemophagocytosis in HLH has a clinical/prognostic significance and would warrant a different treatment strategy, as in this case.
